# Antireflux mucosectomy for gastroesophageal reflux disease: efficacy and the mechanism of action

**DOI:** 10.1055/a-2333-5232

**Published:** 2024-07-09

**Authors:** Thijs Kuipers, Renske A.B. Oude Nijhuis, Roos E. Pouw, Albert J. Bredenoord

**Affiliations:** 1 26066 Gastroenterology and Hepatology, Amsterdam UMC Location AMC, Amsterdam, Netherlands; 2 571165 Gastroenterology, Amsterdam Gastroenterology Endocrinology Metabolism, Amsterdam, Netherlands

## Abstract

**Background** Previous studies suggested that antireflux mucosectomy (ARMS) is effective in reducing reflux symptoms and total acid exposure, although the mechanism is unknown. Our objective was to investigate the effect of ARMS on reflux parameters and its mechanism of action.

**Methods** Gastroesophageal reflux disease (GERD) patients with insufficient symptom control despite a twice-daily proton pump inhibitor (PPI) underwent a piecemeal multiband mucosectomy of 50% of the circumference of the esophagogastric junction (EGJ), extending 2 cm into the cardia. The primary end point was the total number of reflux episodes during 24-hour pH-impedance studies.

**Results** 11 patients (8 men; median age 37 [interquartile range (IQR) 32–57] years) were treated, with one patient subsequently lost to follow-up. ARMS reduced the median (IQR) number of total reflux episodes (74 [60–82] vs. 37 [28–66]; P = 0.008) and total acid exposure time (8.7% [6.4%–12.7%] vs. 5.3% [3.5%–6.7%]; P = 0.03). Treatment reduced the median (IQR) number of transient lower esophageal sphincter relaxations (TLESRs) during a 90-minute postprandial period (4 [1–8] vs. 2 [1–4]; P = 0.03) and reflux symptom scores (3.6 [3.6–3.9] vs. 1.6 [0.7–2.7]; *P* = 0.005). Treatment did not increase the mean (SD) dysphagia scores (8.2 [7.3] vs. 8.5 [6.5]) or change the EGJ distensibility on impedance planimetry (4.4 [2.1] vs. 4.3 [2.2] mm2/mmHg). One delayed post-procedural bleed requiring repeat endoscopy occurred (10%); no strictures developed.

**Conclusion** ARMS is an effective treatment option in PPI-refractory GERD, reducing acid exposure, reflux episodes, and symptoms. While its working mechanism could not be explained by a difference in distensibility, a reduction in TLESRs might play a role.

## Abstract (de)

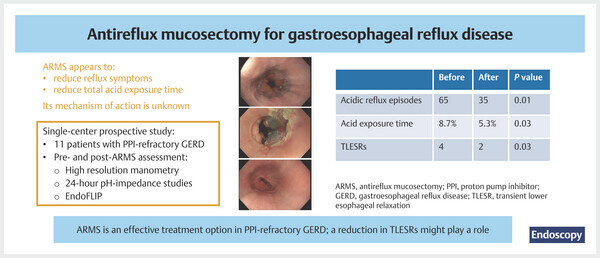

## Introduction

Gastroesophageal reflux disease (GERD) is a common condition where backflow of gastric contents into the esophagus causes esophageal damage and/or bothersome symptoms such as heartburn, regurgitation, and chest pain. Most reflux episodes occur after the meal, when the stomach is full of ingested foods 1 . Gastric distension activates stretch receptors in the proximal stomach and triggers transient lower esophageal sphincter relaxations (TLESRs), which are thought to be the predominant mechanism underlying the postprandial increase in reflux 2 3 .

GERD treatment consists of nonpharmacologic (weight loss, head of bed elevation, abdominal breathing exercises) and pharmacologic options (antacids, H _2_ -receptor blockers, proton pump inhibitors [PPIs]) 4 5 , with PPIs the mainstay in the management of reflux disease. Laparoscopic fundoplication is considered an alternative therapy when pharmacologic treatment fails and is proven to be highly effective 1 . It is however an invasive procedure and therefore is not attractive to all patients with refractory symptoms 6 . Several less invasive endoscopic antireflux procedures have been proposed over the years 7 8 9 ; however, various problems with techniques, costs of equipment, implantation of foreign objects, safety issues, and lack of efficacy have resulted in little enthusiasm for these endoscopic procedures and none has become widely accepted as a standard treatment for reflux disease.

In 2014, antireflux mucosectomy (ARMS), which uses endoscopic mucosal resection (EMR) to resect limited parts of the gastric mucosa along the lesser curve of the cardia, was introduced. First results showed that reflux symptoms resolved in the majority of patients and the mean 24-hour esophageal acid exposure time decreased from 39% to 3% 10 . Subsequently, larger case series with a longer duration of follow-up confirmed that ARMS appears to be an efficacious and feasible procedure, without significant intra- and postoperative morbidity 11 12 . More recently, long-term follow-up results of the ARMS procedure have confirmed these results, with ARMS resulting in a positive effect in 68% of patients at 5-year follow-up 13 .

The available studies have proven the efficacy of the ARMS procedure; however, the reason why ARMS has such a good effect in reducing both esophageal acid exposure and reflux symptoms is unknown. It has been postulated that formation of fibrosis after ARMS constricts and tightens the esophagogastric junction (EGJ) 12 . Partial resection of the cardiac mucosa, as done with ARMS, may also lead to a loss of stretch receptors in the gastric wall, thereby reducing the numbers of TLESRs; however, this hypothesis has never been investigated and the exact effect of ARMS on reflux episodes and the mechanisms through which reflux control is achieved have not yet been elucidated. Therefore our aim was to further study the efficacy of ARMS in patients with reflux and primarily to investigate the underlying mechanism of action through which reflux control is achieved.

## Methods

### Study design

We performed a single-center prospective therapeutic interventional study between December 2019 and September 2023. The local Medical Ethics Committee approved the study (2019_145#B2019587) on 22 August 2019. Written informed consent was obtained from all patients. All authors had access to the complete study data and reviewed and approved the final manuscript.

### Patient selection

We included adults with uncomplicated confirmed GERD (24-hour ambulatory pH-impedance study with a symptom association probability ≥95%; and esophageal acid exposure ≥4%, measured after PPIs had been discontinued for 7 days) and insufficient symptom control on PPI therapy. The main exclusion criteria were: a hiatal hernia >2 cm and the presence of esophagitis of Los Angeles grade C or D. A list of all of the inclusion and exclusion criteria can be found in **Appendix 1s** (see online-only Supplementary Material) *.*

### Study protocol

High resolution manometry (HRM) and 24-hour pH-impedance studies were performed to confirm GERD and to rule out other esophageal diseases. The additional esophageal function studies were performed prior to treatment with ARMS and 3 months after treatment. An upper gastrointestinal (GI) endoscopy was also performed 3 months after treatment to assess healing and the presence of strictures and esophagitis. An overview of the study visits can be found in **Table 1s** .

### Medication

Antisecretory medication was discontinued at least 7 days prior to the study investigations (esophageal function tests and ARMS procedure). Antacids (maximum of six a day) were permitted as rescue medication except on the day of investigation. PPIs were restarted on the day of the ARMS procedure and continued for 1 month afterward, being gradually tapered off over 1 week and then discontinued. If GERD symptoms returned after discontinuation of PPI therapy, a stepwise rescue therapy was implemented, starting with antacids (maximum six tablets daily). If symptoms persisted, PPIs were reinstituted at the initial dose.

### Study procedures

#### Stationary studies

Stationary esophageal HRM was performed, according to the standardized protocol used in our center, to evaluate esophageal motility with the patient in the supine position. Subsequently, the pH-impedance catheter was introduced while leaving the HRM catheter in situ. Patients consumed a standardized meal (one Quarter Pounder and 200 mL orange juice; total 625 Kcal) while in an upright position within 30 minutes. After completion of the meal, a 90-minute postprandial period of pressure, pH, and impedance recording was performed in the supine position. The occurrence of complete TLESRs was analyzed during the postprandial period according to validated criteria 14 .

#### Ambulatory 24-hour pH-impedance study

Thereafter, the manometry catheter was removed and the 24-hour ambulatory pH-impedance catheter was left in the esophagus, with analysis done according to the Lyon consensus 15 .

#### Impedance planimetry

Prior to the stationary esophageal studies, an impedance planimetry study was performed to assess the EGJ distensibility (EndoFLIP catheter, model EF-325N; Crospon Ltd., Galway, Ireland). The center of the bag was positioned at the EGJ and the bag was inflated by the following distension protocol: 20-mL, 30-mL, 40-mL, and 50-mL volumes. A volume of 40 mL was used to assess the EGJ distensibility, with EGJ distensibility expressed in mm ^2^ /mmHg 16 . At this volume, 95% of normal subjects will have a EGJ distensibility above 2 mm ^2^ /mmHg; values below are considered abnormal 17 .

#### ARMS procedure

Video showing the antireflux mucosectomy (ARMS) procedure being performed.Video 1

ARMS was performed, as described by Inoue et al. 10 , in patients under deep propofol sedation. During upper GI endoscopy, EMR of the EGJ mucosa was conducted in a piecemeal fashion using Multiband Mucosectomy (Duette; Cook, Limerick, Ireland), with prior submucosal lifting using a mixture of saline, adrenaline, and indigo carmine. A forward-viewing upper GI endoscope (GIF HQ190; Olympus, Hamburg, Germany) was used.

First, the scheduled reduction area was marked on the mucosa, with markings placed along the expected margin of the EMR using an electrocautery knife connected to an electrocautery generator (Erbe Vio 300D; Erbe Elektromedizin, Tübingen, Germany). The total mucosectomy was approximately 3 cm in length (1 cm in the esophagus and approximately 2 cm in the stomach). Instead of the two-thirds as described by Inoue et al. 10 , we treated only 50% of the circumference of the EGJ at the lesser curvature side, in order to reduce the risk of dysphagia. EMR was carried out repeatedly until the marked mucosal area had been completely resected. A coagulating forceps (FD-410LR Coagrasper; Olympus) was used for hemostasis if needed ( Video 1 ).

Patients were kept on a clear liquid diet for 12 hours. Assuming there were no postoperative alarm symptoms, patients were discharged on the day of the procedure, with the continuation of twice daily PPIs for 4 weeks.

### Statistical analysis

#### Sample size

We based our sample size calculation on a previous study in which a similar population was studied 18 . In a group of patients with GERD confirmed by 24-hour ambulatory pH-impedance studies, the mean total number of reflux episodes was 97.6 (SD 31). A 30% decrease in reflux episodes (68.3 episodes) was considered clinically relevant. Based on these numbers and a paired two-sided *t* test with a significance level of 5% and a power of 80%, a sample size of 11 subjects was calculated as being required.

#### End point analysis

The primary outcome was a post-treatment change in reflux episodes during 24-hour pH-impedance measurement. Secondary outcomes included: the acid exposure time, number of reflux episodes (acidic, weakly acidic, and gaseous) and belching (gastric and supragastric); manometric features; EGJ distensibility; number of TLESRs; reflux symptoms, dysphagia symptoms, health-related quality of life ( **Appendix 2s** ), and PPI use in the preceding month (PPI use in the week prior to investigation was excluded as PPIs were stopped in this week); grade of gastroesophageal flap valve according to the Hill classification and erosive esophagitis according the Los Angeles classification assessed during endoscopy; and occurrence of unwanted procedure-related events including perforation, (delayed) bleeding, and strictures.

Continuous data were compared with the paired Student’s *t* test for normally distributed data and the Wilcoxon signed rank test for non-normally distributed data. Categorical variables were evaluated using McNemar’s test. Descriptive statistics were presented as percentage, mean (SD), or median (interquartile range [IQR]). Correlations were evaluated using Pearson’s correlation. A *P* value of <0.05 was considered significant. SPSS statistics (version 28; SPSS) was used for the statistical analysis.

## Results

**Fig. 1:**
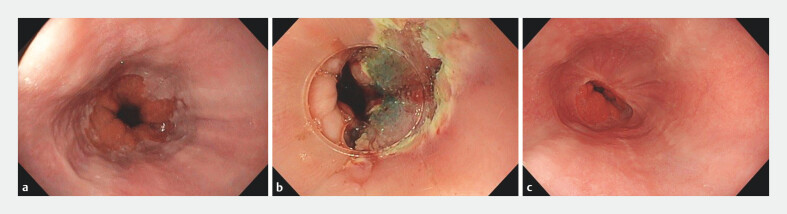
Endoscopy images: **a** prior to treatment with antireflux mucosectomy (ARMS); **b** during the ARMS procedure; **c** 3 months after treatment with ARMS.

In total, 15 patients signed the informed consent form, with 11 patients treated (8 men [73%]). The reasons for exclusion are shown in **Fig. 1s** *.* One patient was lost to follow-up and therefore was not included in further analysis apart from the baseline criteria ( Table 1 ). Example endoscopy images prior to, during, and after treatment can be found in Fig. 1 .

**Table:** **Table 1** Baseline characteristics of the 11 patients included in the study.

	n (%), unless otherwise stated
Age, median (IQR), years	37 (32–57)
Sex, male	8 (73%)
BMI, mean (SD), kg/m ^2^	28.4 (3.5)
Current smoker	2 (18%)
Alcohol consumption	
No	4 (36%)
Mild (0–5 U/week)	6 (55%)
Moderate (5–14 U/week)	1 (9%)
Medication use	
Antacids	2 (18%)
H2-receptor antagonists	1 (9%)
Proton pump inhibitors	11 (100%)
High resolution manometry diagnosis	
Normal	3 (27%)
Ineffective esophageal motility	8 (73%)
Questionnaire results, median (IQR)	
Reflux disease questionnaire (RDQ-GERD)	3.5 (2.1–3.9)
GERD health-related quality of life	28 (21–32)
Brief dysphagia questionnaire	7 (1–14)
GERD, gastroesophageal reflux disease; IQR, interquartile range.	

### 24-hour ambulatory pH-impedance monitoring

**Fig. 2:**
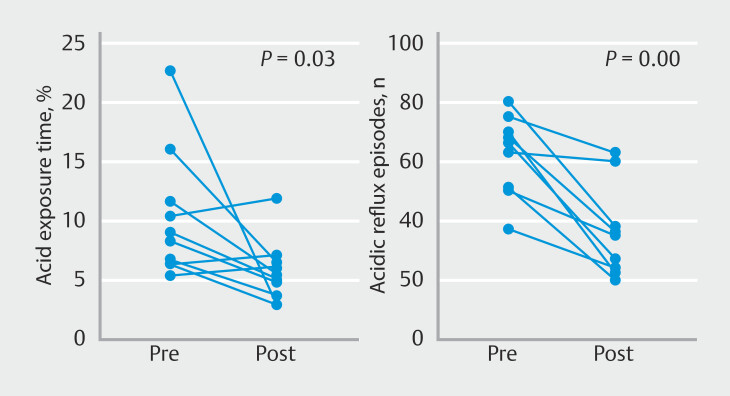
Charts showing the changes measured during 24-hour pH-impedance measurement for: **a** total acid exposure time; **b** acidic reflux episodes.

The 24-hour pH-impedance monitoring revealed a significant reduction in the total acid exposure time (median 8.7% [IQR 6.4%–12.7%] vs. 5.3% [3.5%–6.7%]; *P* = 0.03), as shown in Fig. 2 . In addition, the median (IQR) total number of reflux episodes (74 [60–82] vs. 37 [28–66]; *P* = 0.008) and number of acidic reflux episodes (65 [50–71] vs. 35 [23–49]; *P* = 0.008) decreased significantly after treatment; no significant difference was seen in weakly acidic reflux episodes (9 [4–16] vs. 4 [3–6]; *P* = 0.05). The median (IQR) number of gastric belches (42 [22–56] vs. 43 [17–46]; *P* = 0.14) and supragastric belches (7 [2–85] vs. 5 [4–29]; *P* = 0.44) did not change significantly after treatment.

### 90-minute postprandial stationary measurement

**Fig. 3:**
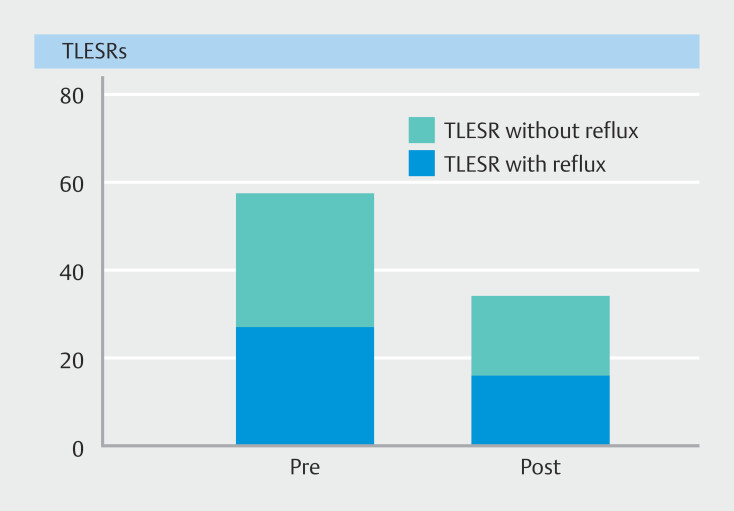
Bar chart of the total number of complete transient lower esophageal sphincter relaxations (TLESRs) with and without associated reflux seen during the 90-minute postprandial measurement period in all subjects pre- and post-treatment.

During the 90-minute postprandial measurement period, we found a significant decrease in the median (IQR) complete TLESRs after intervention compared with baseline (4 [1–8] vs. 2 [1–4]; *P* = 0.03). In all patients combined, a total of 58 complete TLESRs were found prior to treatment, 27 (47%) of which were associated with reflux, compared with a total of 34 complete TLESRs after treatment, with 16 (47%) associated with reflux ( Fig. 3 ).

We did not see a significant difference in the median (IQR) total number of reflux episodes (9 [3–13] vs. 6 [4–12]; *P* = 0.80), acidic reflux episodes (9 [2–12] vs. 5 [4–10]; *P* = 0.72), or weakly acidic reflux episodes (0 [0–0] vs. 0 [0–1]; *P* = 0.68) during the postprandial measurement period compared with baseline. In addition, no significant difference was seen in the median (IQR) total acid exposure time (15.1% [5.2%–47.8%] at baseline vs. 20.8% [3.2%–29.1%] after treatment; *P* = 0.80).

### Esophagogastric junction

After ARMS treatment, the median (IQR) LES resting pressure increased significantly from 16.5 (3.3–22.5) mmHg to 18.3 (12.8–39.5) mmHg ( *P* = 0.047). The median (IQR) IRP-4 was also affected by ARMS treatment, although not significantly, increasing from 3.9 (0–10.1) mmHg to 11.5 (5.9–13.9) mmHg ( *P* = 0.09). We did not found a significant change in the mean (SD) EGJ distensibility measured using impedance planimetry (4.4 [2.1] vs. 4.3 [2.2] mm ^2^ /mmHg; *P* = 0.95).

### Questionnaires

After treatment, significantly fewer reflux symptoms were reported based on the RDQ-GERD score (median 3.6 (IQR 3.6–3.9) at baseline vs. 1.6 (0.7–2.7); *P* = 0.005). ARMS treatment resulted in a decrease in the median (IQR) GERD health-related quality of life score (26 (21–32) vs. 16 (6–24); *P* = 0.008), indicating that GERD-related quality of life improved. We did not see a significant change in the mean (SD) dysphagia symptoms (8.2 [7.3] vs. 8.5 [6.5]; *P* = 0.88). An overview of the treatment effects of ARMS can be found in Table 2 .

**Table:** **Table 2** Treatment effects assessed by various different techniques in the 10 patients who underwent antireflux mucosectomy (ARMS).

	Prior to treatment	3 months after treatment	P value
**24-hour ambulatory pH-impedance monitoring**			
Total reflux episodes, median (IQR), n	74 (60–82)	37 (28–66)	0.008
Acidic reflux episodes, median (IQR), n	65 (50–71)	35 (23–49)	0.008
Weakly acidic reflux episodes, median (IQR), n	9 (4–16)	4 (3–6)	0.05
Total acid exposure time, median (IQR), %	8.7 (6.4–12.7)	5.3 (3.5–6.7)	0.03
**High resolution manometry**			
IRP-4, median (IQR), mmHg	3.9 (0–10.1)	11.5 (5.9–13.9)	0.09
LES resting pressure, median (IQR), mmHg	16.5 (3.3–22.5)	18.3 (12.8–39.5)	0.047
**90-minute postprandial manometry and pH impedance**			
TLESRs, median (IQR), n	4 (1–8)	2 (1–4)	0.03
Total reflux episodes, median (IQR), n	9 (3–13)	6 (4–12)	0.80
Acidic reflux episodes, median (IQR), n	9 (2–12)	5 (4–10)	0.72
Weakly acidic reflux episodes, median (IQR), n	0 (0–0)	0 (0–1)	0.68
Total acid exposure time, median (IQR), %	15.1 (5.2–47.8)	20.8 (3.2–29.1)	0.80
**EndoFLIP**			
Distensibility index (DI) at 40 mL, mean (SD), mm ^2^ /mmHg	4.4 (2.1)	4.3 (2.2)	0.95
**Endoscopy**			
Esophagitis present on endoscopy, n (%)	9 (90.0)	5 (50.0)	0.13
Los Angeles grade A / B, n (%)	4 (40.0) / 5 (50.0)	1 (10.0) / 4 (40.0)	
Hill classification during endoscopy, n (%)			
Hill 1	5 (50.0)	6 (60.0)	
Hill 2	3 (30.0)	4 (40.0)	
Hill 3	2 (20.0)	-	
**Questionnaire symptom scores**			
Reflux Disease Questionnaire, median (IQR)	3.6 (3.6–3.9)	1.6 (0.7–2.7)	0.005
GERD Health-Related Quality of Life, median (IQR)	26 (21–32)	16 (6–24)	0.008
Brief Esophageal Dysphagia Questionnaire, mean (SD)	8.2 (7.3)	8.5 (6.5)	0.88
GERD, gastroesophageal reflux disease; IQR, interquartile range; LES, lower esophageal sphincter; TLESR, transient LES relaxation.			

### Medication

Prior to treatment, all patients were using PPIs twice daily. After treatment, 3/10 patients (30%) were able to cease all reflux medication, while 6/10 (60%) were still taking PPIs; however it should be noted that two of these patients (20%) had to take PPI not because of reflux symptoms, but as gastroprotection owing to co-medication. One patient (10%) was using only antacids on a regular basis.

### Post hoc correlations

We explored correlations between reflux episodes and pathophysiologic parameters (IRP-4, LES resting pressure, TLESRs, and EGJ distensibility). After treatment, we found a correlation between the number of TLESRs in the postprandial recording period and the acid exposure time in the supine position during the 24-hour pH-impedance measurement ( *r* = 0.66; *P* = 0.04). We also discovered a correlation between the number of TLESRs and the number of weakly acidic episodes during 24-hour pH-impedance measurement ( *r* = 0.76; *P* = 0.02). Additionally we found an inverse correlation between IRP-4 and the number of weakly acidic reflux episodes measured during the postprandial period ( *r* = −0.65; *P* = 0.04).

### Safety

One delayed post-procedural bleed (10%) occurred, which required repeat endoscopy and readmission for 1 night. During endoscopy, the bleeding had already stopped and no endoscopic intervention was necessary, nor was a transfusion. In one patient (10%), the procedure was terminated owing to sedation-related desaturation, which fully recovered once the patient was awake; this patient was lost to follow-up. No significant esophageal strictures were seen after treatment.

## Discussion

We have evaluated the efficacy, underlying mechanisms, and safety of the ARMS procedure in patients with PPI-refractory reflux symptoms. We found that ARMS resulted in a significant decrease in the number of compete TLESRs and a higher IRP-4 (although not significantly), while the LES resting pressure and the number of belches remained unchanged. Furthermore, we found significant reductions in total acid exposure, total number of reflux episodes, number of acidic reflux episodes, with a trend also visible in the healing of esophagitis. Simultaneously, patients’ GERD-specific quality of life and reflux symptoms improved. In addition, we found correlations between the number of TLESRs and both acid exposure time in the supine position and the number of weakly acidic reflux episodes, suggesting that the reduction in TLESRs after ARMS may be an important driver of the reduction of acid exposure and improved reflux symptoms.

We found a significant decrease in total acid exposure time from 8.7% to 5.3% after treatment. Acid exposure times in earlier studies that performed esophageal 24-hour pH monitoring prior to and after treatment with ARMS have varied widely (20.8%–3.1% prior to treatment and 6.9%–1.8% after treatment); however, all studies found a significant reduction in acid exposure time 19 20 21 22 . Comparing our results to laparoscopic fundoplication, it seems evident that acid exposure time is more markedly reduced after fundoplication (1.8%–0.3%) than after ARMS (5.3%) 23 .

Patients in our study reported a significant reduction of reflux symptoms based on the RDQ-GERD questionnaire (3.6 before treatment vs. 1.6 after treatment). Although most ARMS studies have evaluated symptoms based on the Gastroesophageal Reflux Disease Questionnaire (GERDQ) score (13.3–9.4 before treatment vs. 9–3.4 after treatment), the results of our trial are in line with the previously reported studies 22 .

We did not find a significant change in the number of gastric belches during 24-hour pH-impedance measurement pre- and post-treatment with ARMS (42 vs. 43 belches). This is in contrast to surgical antireflux procedures (laparoscopic fundoplication) where a significant decrease in the number of gastric belches has been seen after treatment (60 vs. 12 belches) 24 . Bloating and inability to belch have been reported after laparoscopic fundoplication and lead to decreased satisfaction with the outcome 24 25 . Because the number of gastric belches are not affected by the ARMS procedure, it appears it may not result in bloating and an inability to belch.

When looking at the safety parameters in our study, one patient (10%) was readmitted owing to delayed bleeding, no perforations occurred, and no strictures were seen at follow-up endoscopy. Other studies have reported the percentage of patients with bleeding to range from 0 to 43%, with a mean of 5% 26 . The total number of patients in these studies ranged from 12 to 109. Because our study population was small (n = 11), the percentage of patients with delayed bleeding may be overvalued compared with the previous studies. We did not see any esophageal strictures, while other studies reported a incidence of 10.6% 26 . This might be explained by the fact that, in our study, only 50% of the circumference around the EGJ was treated, in comparison with 60%–80% of the circumference in other studies 10 22 .

An important objective of this study was to clarify the underlying working mechanism of ARMS. We evaluated the number of complete TLESRs after the ARMS procedure and found that the number of TLESRs during the 90-minute postprandial measurement period was reduced by approximately 50% (from 4 [1–8] to 2 [1–4]). The absolute number of complete TLESRs found may be on the lower side compared with previous studies. Although the variability in frequency of TLESRs has varied from 0 to 12 TLESRs/hour in different studies 27 28 , both studies included both complete and incomplete TLESRs. To our knowledge, ours is the first study to analyze the number of TLESRs after ARMS.

TLESRs are mainly triggered by gastric distension through tension receptors that are located in the subcardiac region of the stomach 29 . Because the mucosectomy is extended into the stomach for 2 cm, it is hypothesized that the gastric stretch receptors have become less sensitive to gastric distension and the number of TLESRs is reduced. It is known that a surgical fundoplication also reduces the number of TLESRs and this reduction is thought to have an important role in reducing reflux episodes and acid exposure time 30 .

In addition, we also found correlations between a decreased number of TLESRs and both decreased number of weakly acidic reflux episodes and reduced acid exposure time during ambulatory pH-impedance measurement, which indicate this might be one of the mechanisms that explain the effect of ARMS. During the postprandial period, no correlation between TLESRs and acid exposure or reflux episodes was seen, but this may be explained by the small number of reflux episodes measured in the 90-minute postprandial period. It is important to note that the sample size of our study is small and therefore some caution is advised when interpreting these correlations.

Another assumed mechanism of action of ARMS is the formation of a mechanical reflux barrier owing to fibrosis at the EGJ. In our study, we did not found a significant change in EGJ distensibility (4.4 vs. 4.3 mm ^2^ /mmHg). Therefore, we could conclude the mechanical barrier formed by fibrosis is not one of the underlying working mechanisms of ARMS; however, it is also possible our sample size was too low to see any effect or the EGJ distensibility protocol was not optimal. In our study, we measured the EGJ distensibility at a balloon volume of 40 mL, while other studies have used different balloon volumes 17 . We did see a trend toward higher IRP-4 after treatment with ARMS (from 3.9 to 11.5 mmHg). This increase was not significant, possibly also owing to the small sample size. In addition, while the effect of the mucosectomy might not result in a change in EGJ distensibility, the fibrosis on the lesser curvature side may still be a mechanical barrier for reflux.

A strength of this study is the fact we focused not only on the effect of ARMS, but also on the underlying working mechanism. Furthermore, participating GERD patients were well characterized and thoroughly studied using different techniques.

Some limitations do however have to be acknowledged. First, we have follow-up only for 3 months after treatment. This seems to be enough to evaluate adverse events such as delayed bleeding (the mean time between EMR and bleeding is 2.5 days) and the occurrence of strictures (mean time between EMR and first dilation for a stricture is 31 days) 31 32 ; however, 3 months might be not enough to evaluate the long-term effect of ARMS on symptoms, acid exposure, and number of reflux episodes. Currently, no long-term (>1 year) data on the effect of ARMS on acid exposure are available. Second, we did not compare ARMS treatment to the current gold standard surgical approach (laparoscopic fundoplication), but the outcome data suggest it is nowhere near as effective. Probably, endoscopic techniques such as ARMS will never be an appropriate alternative for patients who have very severe GERD with a substantial hiatus hernia, but they may play a role for the treatment of patients with a moderate GERD phenotype and an absent-to-small hiatus hernia. Third, our sample size was relatively small for a confirmatory study, although it was suitable to investigate the underlying mechanism of action of ARMS.

The results of this study regarding the effect of ARMS on reflux symptoms and acid exposure are in line with the results that have been published previously. Additionally, we found the effect of ARMS may be driven more by an inhibition of TLESRs than the mechanical reduction of backflow due to scar formation. This could be a point of interest for further studies on endoscopic antireflux treatment. We believe ARMS might have a place as treatment for GERD next to nonpharmacologic (weight loss, head of bed elevation, abdominal breathing exercises), pharmacologic (antacids, H _2_ blockers, proton pump inhibitors), and surgical treatment options.

In conclusion, ARMS is a successful treatment option in PPI-refractory GERD patients, reducing acid exposure, reflux episodes, and symptoms. While the mechanism could not be explained by a difference in EGJ distensibility, a reduction in TLESRs might play a role.

## Supplementary Material

Supplementary Material Zusatzmaterial
