# Supplementary material for: Antireflux mucosectomy for gastroesophageal reflux disease: efficacy and the mechanism of action
Source: Endoscopy. 2024 Jul 9;56(12):897–905. doi: 10.1055/a-2333-5232 (PMC11716543; doi:10.1055/a-2333-5232)
Supplement: Supplementary file 2 — Supplementary Material [file 10-1055-a-2333-5232_23504514.pdf]

Supplementary material

Anti-reflux mucosectomy for gastroesophageal reflux disease: efficacy and the mechanism of action

Thijs Kuipers, Renske A.B. Oude Nijhuis, Roos E. Pouw, Albert J. Bredenoord

Appendix 1s Full list of inclusion and exclusion criteria

Inclusion criteria

- Indication for surgical treatment, defined by objectively confirmed gastroesophageal reflux disease (24-h ambulatory pH-impedance study with a symptom association probability ≥95%; and esophageal acid exposure ≥4%)
- Symptoms of heartburn, regurgitation and/or chest pain under PPI-treatment for at least 3 months at least 3 times a week.
- Use of proton pump inhibitors at a standard dose twice a day for for a period of at least 4 weeks prior to inclusion.

Exclusion criteria

A potential subject who meets any of the following criteria will be excluded from participation in this study:

- ASA classification of III or higher.
- Previous (surgical or endoscopic) anti-reflux procedure
- Previous surgery of the stomach or esophagus
- Sliding hiatal hernia >2cm
- Esophagitis grade C or D
- Presence of Barrett's esophagus with dysplasia
- Known coagulopathy
- Unable to stop coagulants (with the exception of mono antiplatelet therapy)
- Presence of liver cirrhosis and/or esophageal varices
- Presence of a stricture of the esophagus
- Presence of eosinophilic esophagitis
- Presence of achalasia
- Presence of connective tissue disorder
- Absent peristalsis on high-resolution manometry
- Pregnancy at time of treatment

Supplementary material

Table 1s Details of investigations at each visit.

Visit 1<br>*Baseline investigations*	Visit 2	Visit 3<br>*ARMS procedure*	Visit 4<br>*Investigations at 3 months follow-up*	Visit 5
- Informed consent<br>- Medical history<br>- Questionnaires<br>- Esophageal function studies	Removal pH-impedance catheter	Upper endoscopy with ARMS	- Upper endoscopy<br>- Questionnaires<br>- Esophageal function studies	Removal pH-impedance catheter

Appendix 2s Questionnaires

Reflux Disease Questionnaire (RDQ) [1]

A standardized validated questionnaire will be filled in: the reflux-disease questionnaire (RDQ). The RDQ is a 12-item questionnaire assessing the current severity and frequency of 3 GERD-related symptom domains (heartburn, regurgitation and epigastric pain). Each domain is assessed by four questions, all rated on a 5-point Likert scale.

Health-related quality of life for GERD (GERD-HRQL) [2]

GERD-related quality of life (QoL) will be assessed using the GERD-HRQL. This questionnaire was developed to survey symptomatic outcomes and therapeutic effects in patients with GERD. The scale has 11 items, which focus on heartburn symptoms, dysphagia, medication effects and the patient's present health condition. Each item is scored on a 5-point Likert score, with a higher score indicating a better QoL.

Brief Esophageal Dysphagia Questionnaire [3]

A standardized validated questionnaire will be used to assess dysphagia symptoms. The Brief Esophageal Dysphagia Questionnaire (BEDQ) is a 10-item questionnaire assessing both frequency and severity of dysphagia symptoms. The total score is calculated by summing the numeric value (0-5) for all items checked on the questionnaire.

Supplementary material

Fig 1s Study flow chart.

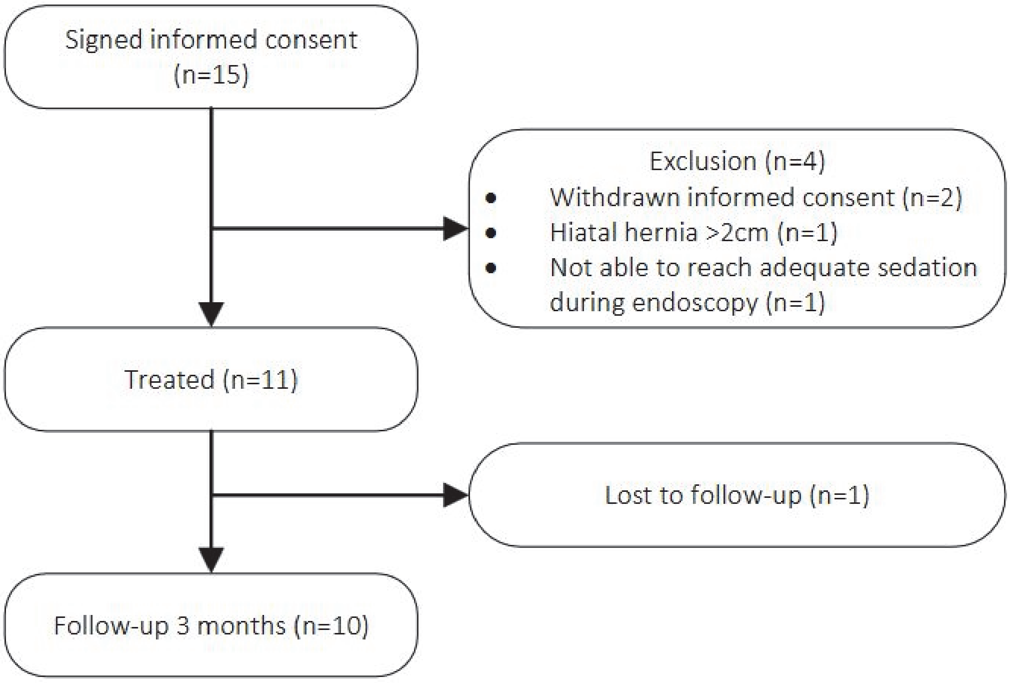

Supplementary References

1. Shaw MJ, Talley NJ, Beebe TJ, Rockwood T, Carlsson R, Adlis S, et al. Initial validation of a diagnostic questionnaire for gastroesophageal reflux disease. Am J Gastroenterol. 2001;96(1):52-7.
2. Velanovich V, Vallance SR, Gusz JR, Tapia FV, Harkabus MA. Quality of life scale for gastroesophageal reflux disease. Journal of the American College of Surgeons. 1996;183(3):217-24.
3. Taft TH, Riehl M, Sodikoff JB, Kahrilas PJ, Keefer L, Doerfler B, Pandolfino JE. Development and validation of the brief esophageal dysphagia questionnaire. Neurogastroenterol Motil. 2016;28(12):1854-60.
